# Supplementary material for: Exceptional Light Propagation via Generalized Bulk‐Edge Correspondence
Source: Nanophotonics. 2026 Jul 2;15(13):e70168. doi: 10.1002/nap2.70168 (PMC13642537; doi:10.1002/nap2.70168)
Supplement: Supplementary file 1 [file NAP2-15-e70168-s001.pdf]

Supporting Information for "Exceptional light propagation via generalized bulk–edge correspondence"

Heitor da Silva,*,† Sergey K. Ivanov,† Isaac Suárez,† José R. Salgueiro,‡ and Albert Ferrando*,†

†*Instituto de Ciencia de los Materiales, Universidad de Valencia, Catedrático J. Beltrán, 2, Paterna, 46980, Spain*

‡*Instituto de Física e Ciencias Aeroespaciais (IFCAE), Universidade de Vigo, Campus de As Lagoas, E-32004 Ourense, Spain*

E-mail: heitor.silva@uv.es; albert.ferrando@uv.es

Contents

1 Wave equations for H and E 3

2 Chern number calculation of the bulk structure 5

3 Frequency dependency on dimerization parameter: tuning knob for system's topology 7

4 Cutoff frequencies for edge modes in topological photonic crystals 8

5 Propagating states in a 1D SSH potential in a 2+1 Schrödinger equation 9

6 Localization–delocalization transition for topological edge modes 14

7 Effective 1+1 Hamiltonian model for L and R edge modes 18

8 Optical dispersion parameters 21

References 23

1 Wave equations for H and E

We present the wave equations solved for the electric and magnetic fields, **H** and **E**, in both periodic (infinite) and truncated (finite) photonic crystal structures. We begin with Eq. (1) of the Main Text, whose general solution for the magnetic field is

$$\mathbf{H}_{n,\mathbf{k}}(\mathbf{r}) = e^{i\mathbf{k}_{\parallel}\cdot\boldsymbol{\rho}}\, e^{i\mathbf{k}_{\perp}\cdot\mathbf{r}_{\perp}}\, \mathbf{u}_{n,\mathbf{k}}(\mathbf{r})\,. \tag{S1}$$

Here, $\mathbf{k}_{\parallel}$ is the wave vector in the xy-plane, $\mathbf{k}_{\perp}$ is the component along the modulation direction of the dielectric function $\varepsilon(z)$, and $\boldsymbol{\rho} = x\hat{\mathbf{x}} + y\hat{\mathbf{y}}$, with $\hat{\mathbf{x}}$ and $\hat{\mathbf{y}}$ being the unit vectors along the x and y axes, respectively. As the crystal exhibits continuous translational symmetry in the xy-plane, k_y can take any real value. However, owing to discrete translational symmetry along z, k_z is restricted to the one-dimensional Brillouin zone.

We define

$$\mathbf{k}_{\parallel} = k_y\hat{\jmath}\,, \quad \mathbf{k}_{\perp} = k_z\hat{k}\,. \tag{S2}$$

The envelope $\mathbf{u}_{n,\mathbf{k}}(\mathbf{r})$ depends only on z and has the general form

$$\mathbf{u}_{n,\mathbf{k}}(z) = (u_x(z), u_y(z), 0)\ . \tag{S3}$$

Due to rotational symmetry in the yz-plane, $u_x(z) = u_y(z)$, allowing the simplification

$$\mathbf{u}_{n,\mathbf{k}}(z) = (u_x(z), 0, 0)\ . \tag{S4}$$

We now evaluate the left-hand side of Eq. (1), which reads

$$\nabla \times \left[\frac{1}{\varepsilon(z)}\nabla \times \mathbf{H}_{n,k_y,k_z}\right] = \nabla \times \mathbf{C}\,, \tag{S5}$$

where

$$\mathbf{C} \equiv \frac{1}{\varepsilon(z)} \nabla \times \mathbf{H}_{n,k_y,k_z} \,. \tag{S6}$$

Developing the curl, we obtain

$$\mathbf{C} = \frac{e^{ik_y y} e^{ik_z z}}{\varepsilon(z)} \left\{ \left[ik_z u_x(z) + \frac{du_x(z)}{dz} \right] \hat{y} - ik_y u_x(z) \hat{z} \right\} \,. \tag{S7}$$

Taking the curl once more gives

$$\nabla \times \mathbf{C} = \nabla \times \left\{ \frac{e^{ik_y y} e^{ik_z z}}{\varepsilon(z)} \left[\left(ik_z u_x(z) + \frac{du_x(z)}{dz} \right) \hat{y} - ik_y u_x(z) \hat{z} \right] \right\} \,. \tag{S8}$$

Combining this with the right-hand side of Eq. (1) yields

$$k_y^2 u_x(z) - \varepsilon(z) \left[ik_z u_x(z) + \frac{du_x(z)}{dz} \right] \frac{d}{dz} \left[\frac{1}{\varepsilon(z)} \right] + k_z^2 u_x(z) - 2ik_z \frac{du_x(z)}{dz} - \frac{d^2 u_x(z)}{dz^2} = \left(\frac{\omega}{c} \right)^2 \varepsilon(z) u_x(z) \,. \tag{S9}$$

We thus obtain the magnetic wave equation:

$$\frac{d^2 u_x(z)}{dz^2} + 2ik_z \frac{du_x(z)}{dz} + \varepsilon(z) \frac{d}{dz} \left[\frac{1}{\varepsilon(z)} \right] \left(ik_z u_x(z) + \frac{du_x(z)}{dz} \right) + \left[\left(\frac{\omega}{c} \right)^2 \varepsilon(z) - k_y^2 - k_z^2 \right] u_x(z) = 0 \,. \tag{S10}$$

For the electric field, starting from Eq. (2) of the Main Text, we write

$$\mathbf{E}_{n,\mathbf{k}}(\mathbf{r}) = e^{i\mathbf{k}_\parallel \cdot \boldsymbol{\rho}} \, e^{i\mathbf{k}_\perp \cdot \mathbf{r}_\perp} \, \mathbf{v}_{n,\mathbf{k}}(z) \,, \quad \mathbf{v}_{n,\mathbf{k}}(z) = (v_x(z), 0, 0) \,. \tag{S11}$$

Taking the double curl yields the electric wave equation:

$$\frac{d^2 v_x(z)}{dz^2} + 2ik_z \frac{dv_x(z)}{dz} + \left[\left(\frac{\omega}{c} \right)^2 \varepsilon(z) - k_y^2 - k_z^2 \right] v_x(z) = 0 \,. \tag{S12}$$

Equations (S10) and (S12) are solved using the Finite-Element Method (FEM) for the infinite (non-truncated) crystal.[1,2] For finite (truncated) structures, the same equations are

considered with $k_z = 0$ and solved using the Transfer-Matrix Method (TMM).[3] In this case, $\varepsilon(z)$ is taken to be piecewise constant, so that its derivative can be neglected in the TMM simulations.

2 Chern number calculation of the bulk structure

In the Main Text, Fig. 2 shows the normalized frequency as a function of the normalized propagation constant for both TE and TM polarizations. These band structures were obtained by solving Eqs. (1) and (2) for the infinite periodic structure using the FEM. The dimerization parameter was set to $\Delta = 0.025\,\mu$m, which, together with the standard values of d_2 and Λ, yields $d_1 = 0.1$ and $d_3 = 0.2\,\mu$m. This choice meets one of the requirements stated in (i) for the topological regime, that is, $\Delta > 0$. The bands exhibit clear differences between TE and TM polarizations due to the significant refractive index contrast. These differences extend to the slopes and overall shapes of the curves bounding the first and higher-order bands, and they strongly influence the dispersion characteristics.

To complement condition (i), we detail the procedure used to evaluate the topological invariant of the nth bulk bands of the infinite periodic lattice. The fields $\mathbf{E}$ and $\mathbf{H}$ admit the unified Bloch form

$$\mathbf{F}_{n,k_y,k_z}(\mathbf{r}) = \big(f_{n,k_y,k_z}(z),\, 0,\, 0\big)\; e^{ik_y y} e^{ik_z z}, \tag{S13}$$

where n labels the band index and k_z is the Bloch momentum restricted to the first Brillouin zone (BZ), $-\pi/\Lambda \leq k_z < \pi/\Lambda$, with Λ being the lattice period. The function $f(z)$ is periodic and satisfies $f(z+R) = f(z)$ for any integer m, where $R = m\Lambda$. For TM polarization we solve Eq. (1) by setting $\mathbf{F} = \mathbf{E}$ ($f = v_x$), whereas for TE we solve Eq. (2) with $\mathbf{F} = \mathbf{H}$ ($f = u_x$). For a fixed value of k_y, the topological index for the nth band is calculated using the expression,[4–6]

$$C_n = \frac{i}{2\pi} \int_{BZ} \langle f_{n,k_z} | \partial_{k_z} | f_{n,k_z} \rangle dk_z\,. \tag{S14}$$

The numerical calculation of the momentum derivative requires the discretization of k_z in the BZ and the evaluation of their corresponding k_z-discretized $f_{n,k_z}(z)$ Bloch functions. However, all these $f_{n,k_z}(z)$ are defined up to a different phase $e^{i\varphi_{k_z}}$ at every k_z. These different phases are uncorrelated for different k_z due to the independent character of the diagonalization procedure of the eigenvalue equations for the TE and TM Bloch functions at every k_z. As a consequence, φ_{kz} fluctuates randomly with k_z making the calculation of the momentum derivative numerically ill-defined. One solution is to provide an additional condition to connect the φ_{kz} phases for different k_z, i.e., to introduce a gauge fixing condition. Another option is to use a gauge-invariant prescription for the calculation of the Zak phase in a discretized BZ. This is given by the gauge invariant loop,

$$\Gamma_n = \left\langle f_{n,1} e^{-2\pi i z}, f_{n,N} \right\rangle \prod_{i=1}^{N-1} \left\langle f_{n,i+1}, f_{n,i} \right\rangle , \tag{S15}$$

which provides the Zak phase for a discrete set of states: $\Gamma = e^{i\gamma_{Zak}}$.[6] In the continuous limit, this definition also provides the topological invariant as in equation (S14) since in this case $\Gamma_n = e^{iC_n}$.[6] We use this numerical method to evaluate the bulk topological index.

The results obtained can be summarized as

$$C_n = 0, \qquad \forall\, n, k_y \qquad\qquad \text{if } \Delta < 0\,, \tag{S16}$$

$$C_n = \begin{cases} +1, & \text{for } n = 1, 3, 5, ... \\ -1, & \text{for } n = 2, 4, 6, ... \end{cases} \qquad \text{if } \Delta > 0 \ \forall k_y \tag{S17}$$

Note that this result holds for all k_y since for solutions with a dependence on k_y as in (S13), the dependence on k_y in the continuous expressions (S14) and discrete (S15) to calculate the bulk topological index trivially disappears.

3 Frequency dependency on dimerization parameter: tuning knob for system's topology

In this section, we illustrate how the field distribution evolves with the dimerization parameter Δ, a structural quantity that determines the system's topology. For $\Delta \leq 0$, the system is topologically trivial, and all modes remain delocalized. In contrast, for $\Delta > 0$, the system enters a nontrivial phase where topological edge modes can appear within the light-line region. In this regime, both extended (delocalized) and localized edge modes coexist among the guided modes of the structure.

We focus on the TM polarization and solve the wave equation using the TMM for a finite structure with 9 periods, $n_l = 1.60$, $n_h = 3.50$, $d_2 = 0.1\,\mu$m, and $\Lambda = 0.5\,\mu$m. Fig. S1 shows the normalized frequency as a function of the dimensionless dimerization parameter, which acts as a tuning knob for the system's topology, together with the corresponding electric and magnetic field profiles. The case $\Delta = 0$ represents a uniform periodic structure without bond alternation—the critical point where the bulk bandgap closes, analogous to the metallic state in the Peierls instability of electronic systems.[6] At this point, and for $\Delta < 0$, the modes are extended, as illustrated by the blue dots 1 and 2 in panel (a) and their field profiles in panel (b). Conversely, for $\Delta > 0$ the structure becomes topologically nontrivial, characterized by a non-zero winding number and the appearance of edge states at the PhC boundaries, as predicted by the bulk–edge correspondence.[7] These occur in symmetric and antisymmetric pairs—represented by the green and red curves in panel (a)—and are exemplified by the blue dots 3–6 in panels (c) and (d) for $\Delta/\Lambda = 0.02$ and 0.05, respectively.

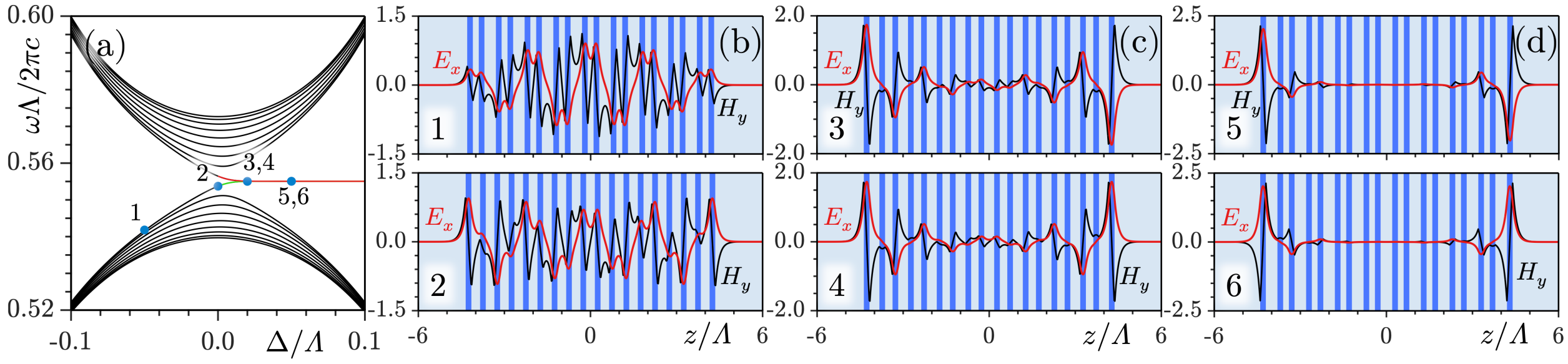

Figure S1: Dependence of the normalized frequency $\omega\Lambda/2\pi c$ on the dimerization parameter Δ/Λ for a finite SSH structure with 9 periods (a) for TM polarization with $k_y\Lambda/2\pi = 1.5$, and the mode profiles (b)–(d) corresponding to the blue dots in panel (a). Panel (b) shows the modes in the trivial regime: the upper plot corresponds to $\Delta/\Lambda = -0.05$ and the lower one to $\Delta/\Lambda = 0.00$. The topological edge modes are presented in panels (c) and (d) for $\Delta/\Lambda = 0.02$ and $\Delta/\Lambda = 0.05$, respectively, for out-of-phase (top) and in-phase (bottom) configurations. The electric field profile E_x is shown in red and the magnetic field profile H_y in black. In panel (a), the green and red branches correspond to the in-phase and out-of-phase topological edge states, respectively. The transition from the trivial to the topological phase occurs at $\Delta/\Lambda = 0$.

4 Cutoff frequencies for edge modes in topological photonic crystals

Dispersion relations of topological edge modes in an SSH photonic crystal must satisfy the condition of lying within the band gap opened inside every conduction band of the bulk when $\Delta > 0$, as shown in Fig. 2 of the Main Text:

$$\omega_-^{\text{band}}(k_y) < \omega_{\text{edge}}(k_y) < \omega_+^{\text{band}}(k_y)\,. \tag{S18}$$

This condition is the same as in the equivalent electronic systems when intercell hopping exceeds intracell hopping, as explained in the previous section. However, as shown again in Fig. 2 of the Main Text, the relativistic nature of Maxwell's equations constrains the dispersion relations of guided modes in *finite* photonic crystal waveguides to lie between two light lines: $\omega_+(k_y) = (c/n_l)k_y$ and $\omega_-(k_y) = (c/n_h)k_y$, which are the linear dispersion relations of a plane wave $\mathbf{E} = \mathbf{E}_0 \exp(ik_y y)$ propagating along the invariant axis y in homogeneous media of refractive indices n_l and n_h, respectively. The relativistic character of the photonic

wave equation is responsible for the linear form of these limiting light lines.

Since the band-gap condition (S18) must be satisfied simultaneously with the guidance condition

$$\omega_-(k_y) \leq \omega_{\rm edge}(k_y) \leq \omega_+(k_y)\,, \tag{S19}$$

the symmetric (S) and antisymmetric (A) guided edge states appearing in a finite topological SSH photonic crystal waveguide must exhibit cutoff frequencies below which they cease to be guided and, therefore, lose localization during propagation. To visualize the existence of these cutoff frequencies, Fig. S2 shows the dispersion relations of the S and A topological edge modes near the upper light line $\omega_+(k_y)$. The change of behavior occurs when k_y fulfills the following crossing conditions:

$$\omega_S(k_y) = \omega_+(k_y) \quad \text{and} \quad \omega_A(k_y) = \omega_+(k_y)\,,$$

which define two threshold values for the wave-vector component, $k_{y,S}$ and $k_{y,A}$. In turn, these threshold values yield the corresponding cutoff frequencies $\omega_{c,S} = \omega_+(k_{y,S})$ and $\omega_{c,A} = \omega_+(k_{y,A})$. Since the dispersion relations of the topological guided modes $\omega_{S,A}(k_y)$ are monotonically increasing functions of k_y, excitation of these modes with frequencies smaller than the cutoff values $\omega_{c,S}$ and $\omega_{c,A}$ will not produce localized edge modes during propagation. In such cases, an initially localized edge mode couples to radiation modes as it propagates, producing losses that cause it to disappear after a finite distance.

5 Propagating states in a 1D SSH potential in a 2+1 Schrödinger equation

Let us now examine the electronic counterpart of the SSH photonic crystal discussed in the Main Text. The physical analogue of this system is a finite periodic array of quantum wells distributed according to the same SSH geometry along the modulation axis z, and invariant

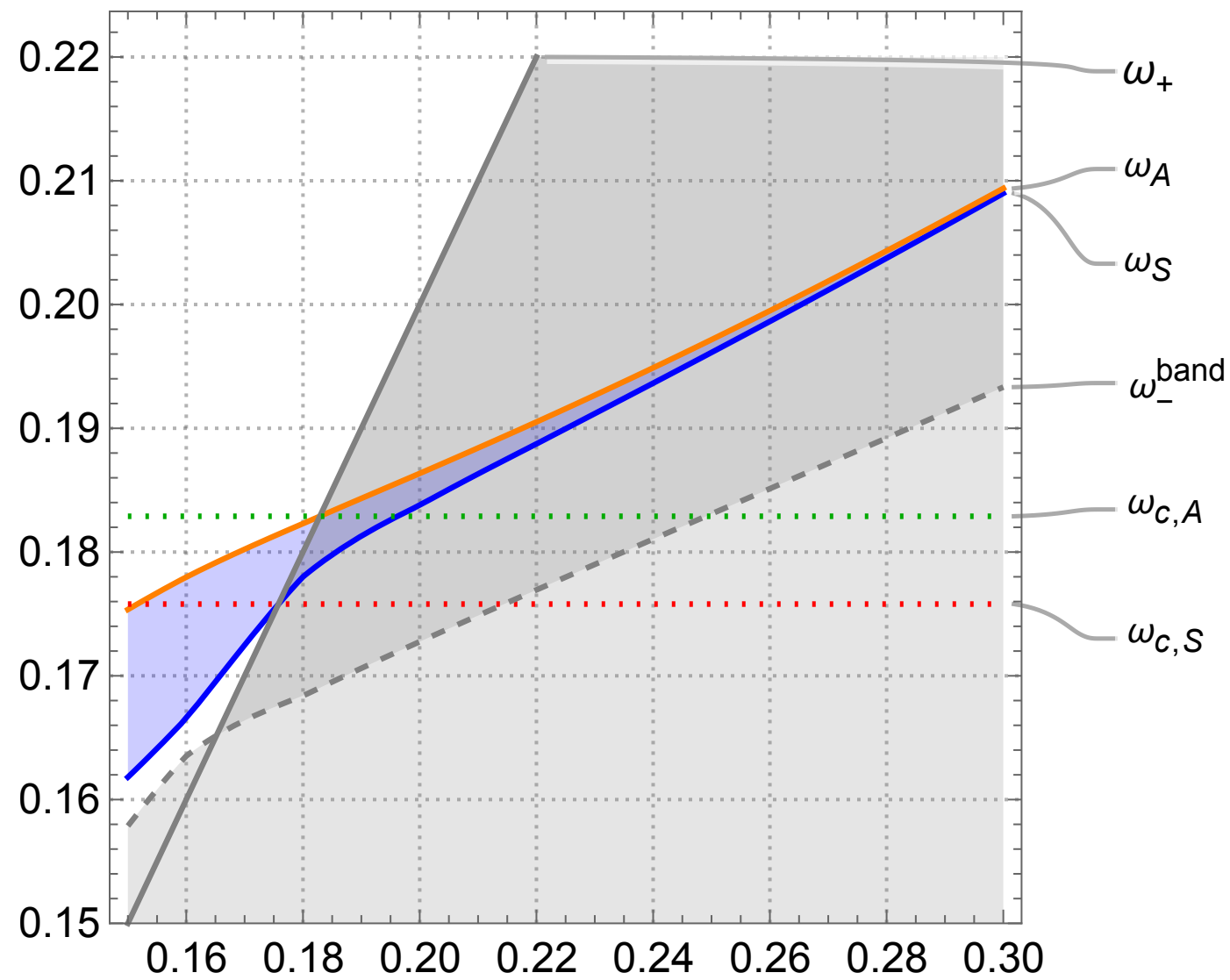

Figure S2: Crossing points of the light line $\omega_+(k_y)$ with the edge-mode dispersion relations $\omega_{S,A}(k_y)$ for a topological SSH waveguide with $\Delta > 0$. Simulation parameters: 9 unit cells, $\Lambda = 1$, $\Delta = 0.1$, $n_l = 1$, and $n_h = \sqrt{13}$.

along the y direction, as illustrated in Fig. 1 of the Main Text. We study the propagation of electronic states along the y axis, whose wave functions are sufficiently broad in the x direction to neglect diffraction in that coordinate. Thus, the single-particle dynamics of the system are governed by a Schrödinger equation in two spatial dimensions (z and y) and time. We recast the standard $2+1$ Schrödinger equation into a dimensionless form by introducing the following variables. For energies and frequencies, we define

$$\bar{E} \equiv \frac{E}{\mathcal{E}} \quad \text{and} \quad \bar{\omega} \equiv \frac{\omega}{\Omega}, \tag{S20}$$

where $\mathcal{E} = \hbar^2/(m\Lambda^2)$ and $\Omega = \hbar/(m\Lambda^2)$ are, respectively, the characteristic energy and frequency determined by the particle mass m and the lattice period Λ. The same length scale Λ is used to introduce the dimensionless coordinates $\bar{y} = y/\Lambda$ and $\bar{z} = z/\Lambda$, as well as the dimensionless wave-vector component along the y-direction, $\bar{k}y = ky\Lambda/(2\pi)$. Finally, using the characteristic frequency Ω, we define a dimensionless time $\bar{t} = \Omega t/(2\pi)$. The final

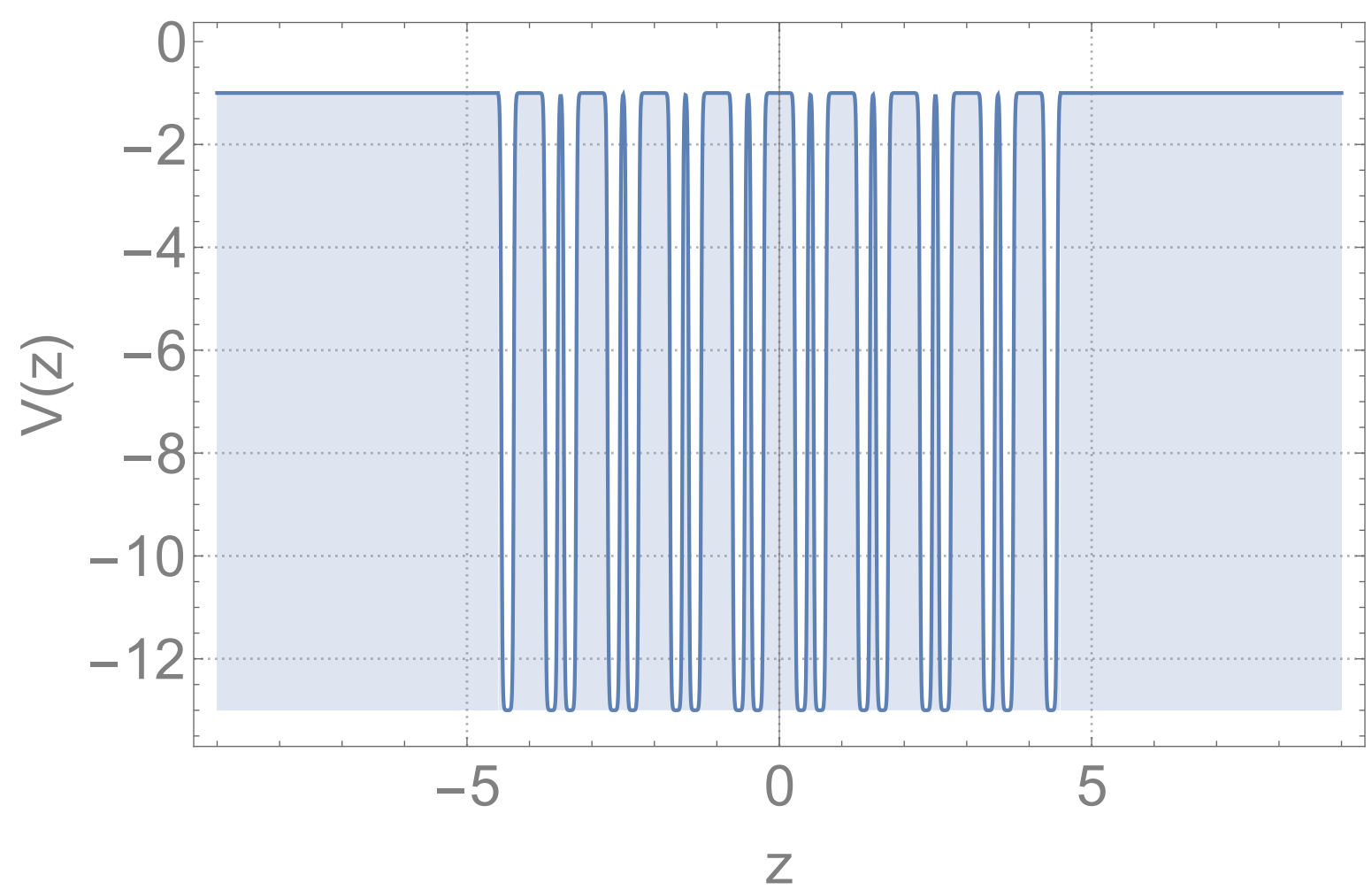

Figure S3: Example of a potential describing a periodic array of quantum wells in a topological SSH configuration. In this case, the particular values defining it are: maximum potential $V_0 = -1$, minimum potential $V_1 = -13$, number of unit cells $n_{\text{cells}} = 9$, lattice period $\Lambda = 1$, dimerization parameter $\Delta = 0.1$, and well width $d_2 = 0.2$.

result for the dimensionless Schrödinger equation is

$$i\frac{\partial}{\partial t}\psi\left(y,z,t\right) = \left[-\frac{1}{2}\left(\frac{\partial^2}{\partial y^2} + \frac{\partial^2}{\partial z^2}\right) + V\left(z\right)\right]\psi\left(y,z,t\right),$$

where, for simplicity, all quantities are assumed to be dimensionless, although the original notation is retained. The potential V is likewise dimensionless and normalized to $\mathcal{E}$.

To model a finite one-dimensional array with a typical topological SSH geometry, we adopt the potential shown in Fig. S3, which represents the counterpart of the dielectric profile of the SSH topological photonic crystal analyzed in the Main Text, such that $V(z) \leftrightarrow -\varepsilon(z) = -n^2(z)$. The maximum and minimum values of the potential, V_0 and V_1, are chosen to correspond to those of its photonic analogue.

In the Main Text, different refractive index values were employed; here, for illustrative purposes, we set $V_0 = -\varepsilon_l = -1$ $(n_l = 1)$ and $V_1 = -\varepsilon_h = -13$ $(n_h = \sqrt{13})$ to enable a direct conceptual comparison between the electronic and photonic cases.

Stationary states are obtained by assuming a harmonic dependence on time and on y in ψ. The latter dependence arises from the translational invariance of the system along

y, since V depends only on z. We therefore search for solutions of the form $\psi(y,z,t;\omega) = \phi(z,\omega)\exp(-i(\omega t - k_y y))$, to obtain the eigenvalue equation

$$\left[\frac{1}{2}k_y^2 + \left(-\frac{1}{2}\frac{\partial^2}{\partial z^2} + V(z)\right)\right]\phi(z,\omega) = \omega(k_y)\ \phi(z,\omega)\,. \tag{S21}$$

Note that, according to Eq. (S20), the dimensionless frequency equals the dimensionless energy, since $E = \hbar\omega$ and $\mathcal{E} = \hbar\Omega$. This eigenvalue equation for stationary states shows that the dependence on k_y of the dimensionless frequency or energy is trivial and identical for all eigenstates:

$$\omega_n(k_y) = \omega_n(0) + \frac{1}{2}k_y^2 \Leftrightarrow E_n(k_y) = E_n(0) + \frac{1}{2}k_y^2.$$

As mentioned in the Main Text, this universal quadratic dependence on k_y arises from the Galilean invariance of the non-relativistic Schrödinger equation. Consequently, the energy gaps between modes at any k_y are identical to those in the spectrum at rest ($k_y = 0$). This feature gives rise to the characteristic stratification of the modal dispersion relations $\omega_n(k_y)$, shown in Fig. S4. This figure corresponds to the potential profile in Fig. S3 with $V_0 = -1$ and $V_1 = -13$, which, as mentioned earlier, match the dielectric constants of the topological photonic structure analyzed in the following sections.

The fact that k_y scaling is trivial has strong implications for the localization of the edge modes in the topological phase of the SSH array, when the dimerization parameter is positive ($\Delta > 0$). As shown in Fig. S4, the two topological edge modes are nearly degenerate (two overlapping light green curves in the figure) and lie in the bound-state region of the spectrum (grey shaded area) for all values of k_y. Bound states at rest ($k_y = 0$; see Eq. S21) belong to the discrete part of the spectrum and must satisfy the well-known condition $v_1 < \omega_{\text{bound}} < v_0$. This condition holds for any k_y, since the spectral gaps remain unchanged (the stratification property). The same argument applies to the bandgap where the topological edge states exist in the topological phase of the SSH potential. Hence, once edge states exist at $k_y = 0$ through the topological mechanism of the SSH potential when $\Delta > 0$, they must remain

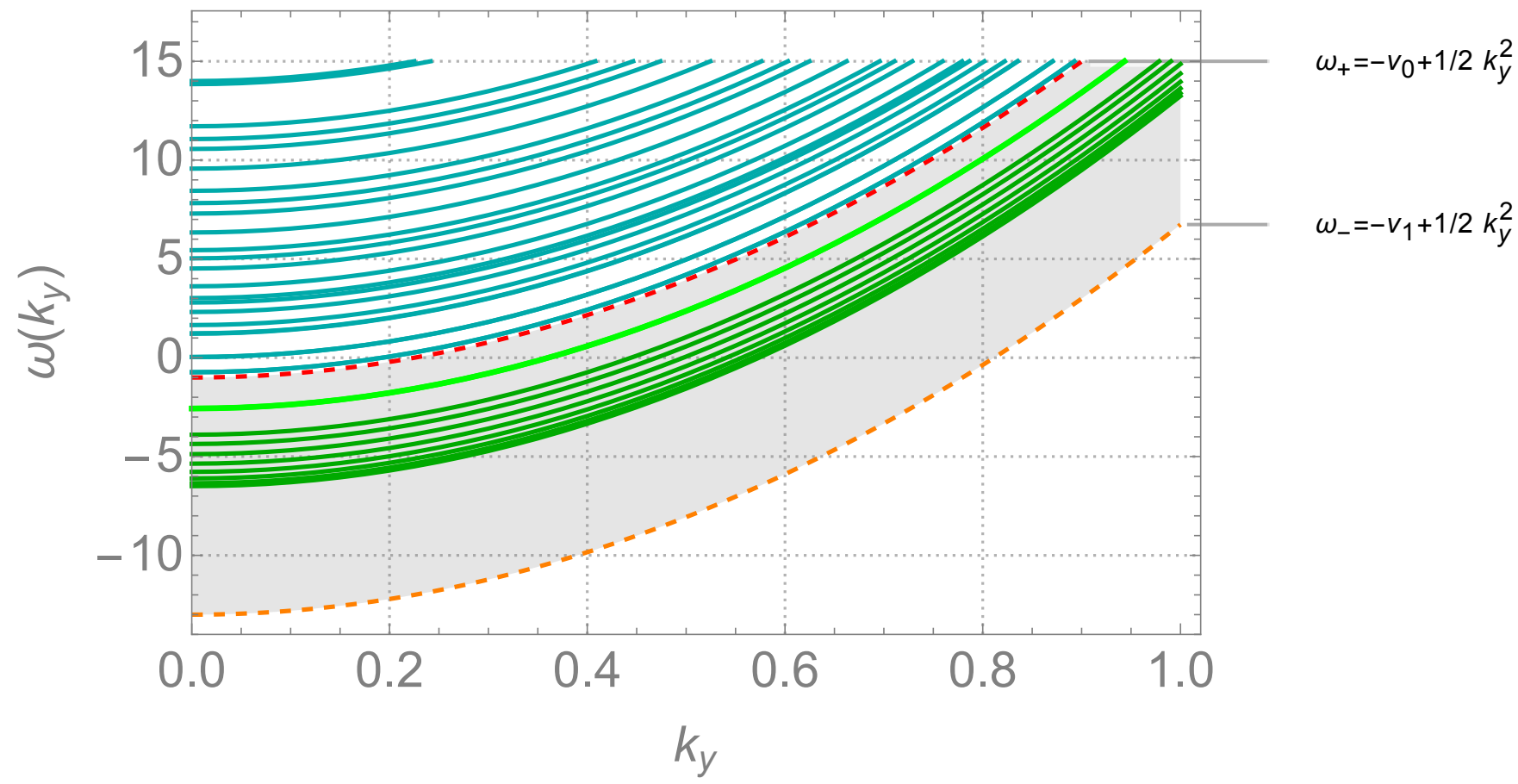

Figure S4: Dispersion relations of propagating states for an electronic system, calculated for the potential in Fig. S3. This potential has the same geometry as the SSH photonic waveguide in Fig. 1 of the Main Text. The dispersion relations of the symmetric and antisymmetric topological edge modes are displayed in light green. The grey shaded region contains the bound (localized) states of the spectrum.

bound and localized for all k_y. Equivalently, they remain edge-localized for all frequencies (or energies) greater than their frequency at $k_y = 0$.

Mathematically, the triviality of edge localization with respect to k_y scaling becomes evident when evaluating the probability density of the edge states using Eq. (S21). The solution of this equation at $k_y \neq 0$ is identical to that at $k_y = 0$: $\phi\left(z, \omega\left(k_y\right)\right) = \phi\left(z, \omega\left(0\right)\right)$, so that the probability density of an edge state, $\left|\phi_{\text{edge}}\left(z, \omega\right)\right|^2 = \left|\phi_{\text{edge}}\left(z, \omega_{\text{edge}}\left(0\right)\right)\right|^2$, remains unchanged for any frequency greater than $\omega\left(0\right)$. Hence, edge localization depends solely on the standard SSH mechanism ($\Delta > 0$) and is k_y-independent, or equivalently, frequency-independent for $\omega \geq \omega_{\text{edge}}\left(0\right)$. This behavior is clearly illustrated in Fig. S5 for the symmetric and antisymmetric edge states.

As far as the bulk index is concerned, the trivial dependence of all propagating eigenmodes on k_y also applies to the Bloch functions: $u_{k_y k_z}(y, z) = u_{k_z}(z) e^{i k_y y}$. In this way, the evaluation of the Berry connection $i\langle u_{k_y k_z} | \partial_{k_z} u_{k_y k_z} \rangle$ remains trivially unchanged for all k_y. The bulk index $\mathcal{I}_b$, which equals the Chern number, as calculated with the general formula (S14), becomes then k_y-independent as well.

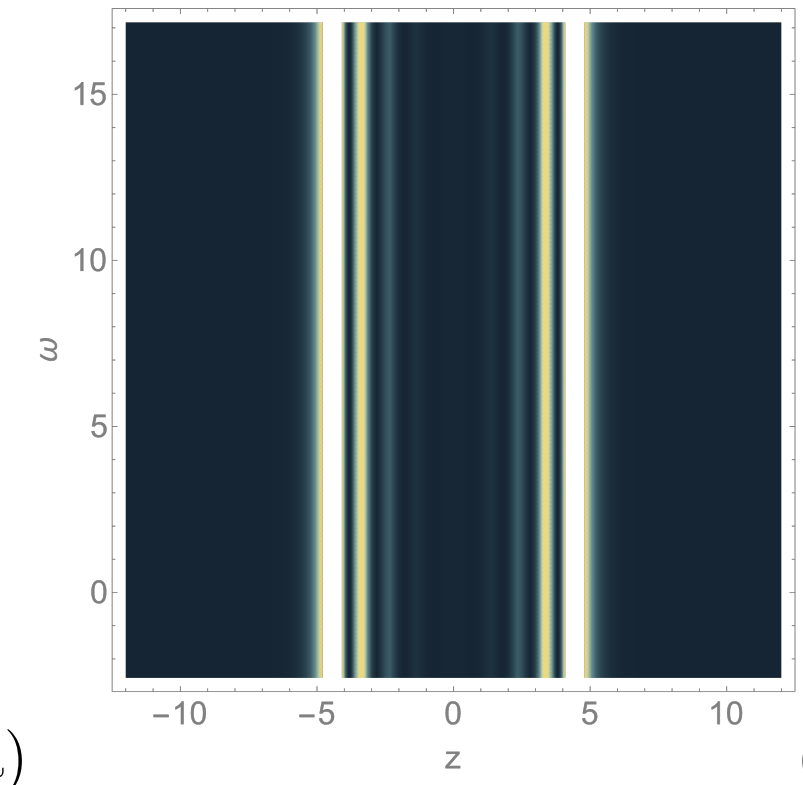

(a)

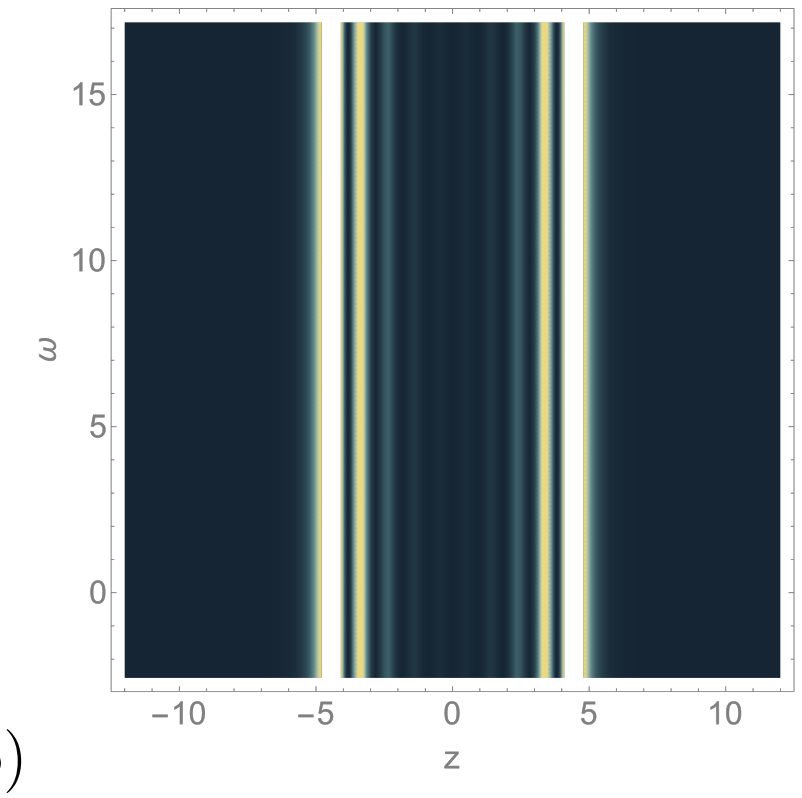

(b)

Figure S5: Spatial distribution of the probability density at different frequencies $|\phi(z,\omega)|$ for (a) the symmetric and (b) antisymmetric edge modes of the SSH potential in Fig. S3. The probability density remains independent of ω (and so does edge localization) for values greater than $\omega_{\text{edge}}(0)$.

6 Localization–delocalization transition for topological edge modes

The existence of cutoff frequencies for the S and A edge modes gives rise to two distinct edge-delocalization mechanisms. The first occurs when the guidance condition (S19) is not fulfilled and, consequently, guidance is lost. As discussed in the previous section, delocalization in this case is produced by radiative losses into the external medium. However, this is not the only delocalization mechanism for edge modes in a finite SSH photonic crystal. This process can be quantified by calculating the localization fractions of electric energy in air, bulk, and edge regions, respectively, as a function of ω:

$$\chi_i^{S,A}(\omega) = \frac{\int_{\Omega_i} dz\, \frac{1}{2}\varepsilon(z) \left|E_x^{S,A}(z,\omega)\right|^2}{U_{\text{tot}}}, \quad i = \text{air, bulk, edges},$$

where Ω_i denotes the spatial domain corresponding to region i, and U_{tot} is the total electric energy in the full domain $\Omega = \Omega_{\text{air}} \cup \Omega_{\text{bulk}} \cup \Omega_{\text{edges}}$. Figure S6 shows the behavior of these localization fractions as a function of ω.

The evolution of $\chi_{\text{edges}}(\omega)$ reveals how edge localization develops progressively from non-existent to dominant as the frequency exceeds the cutoff. This smooth localization process is

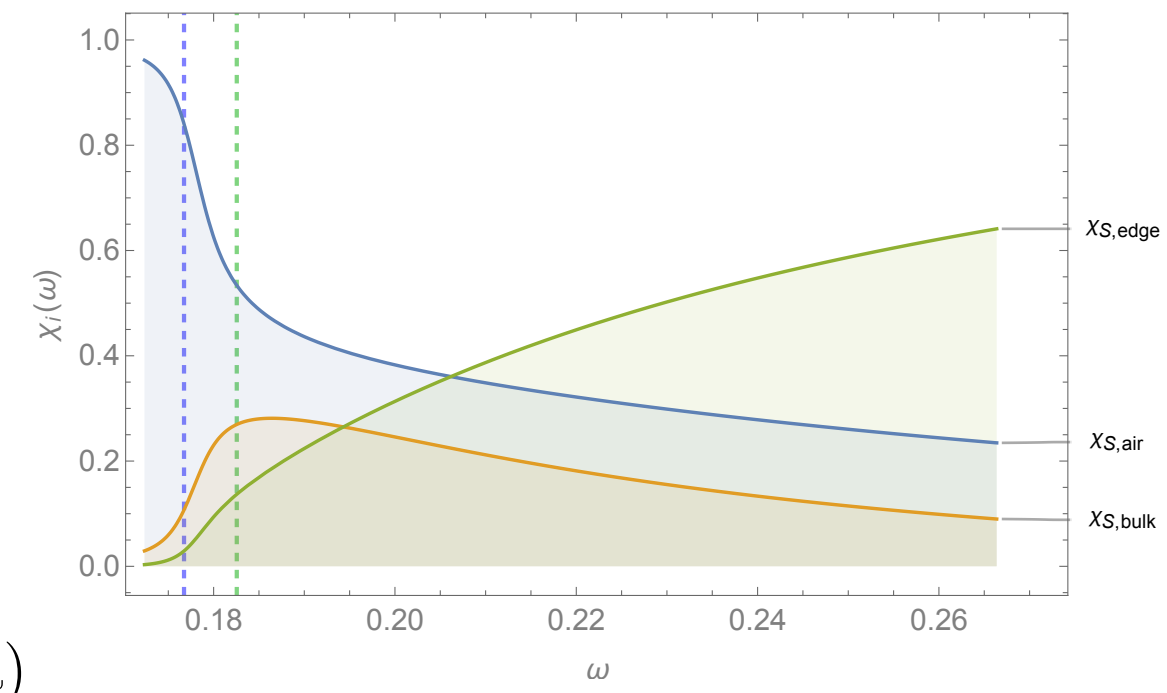

(a)

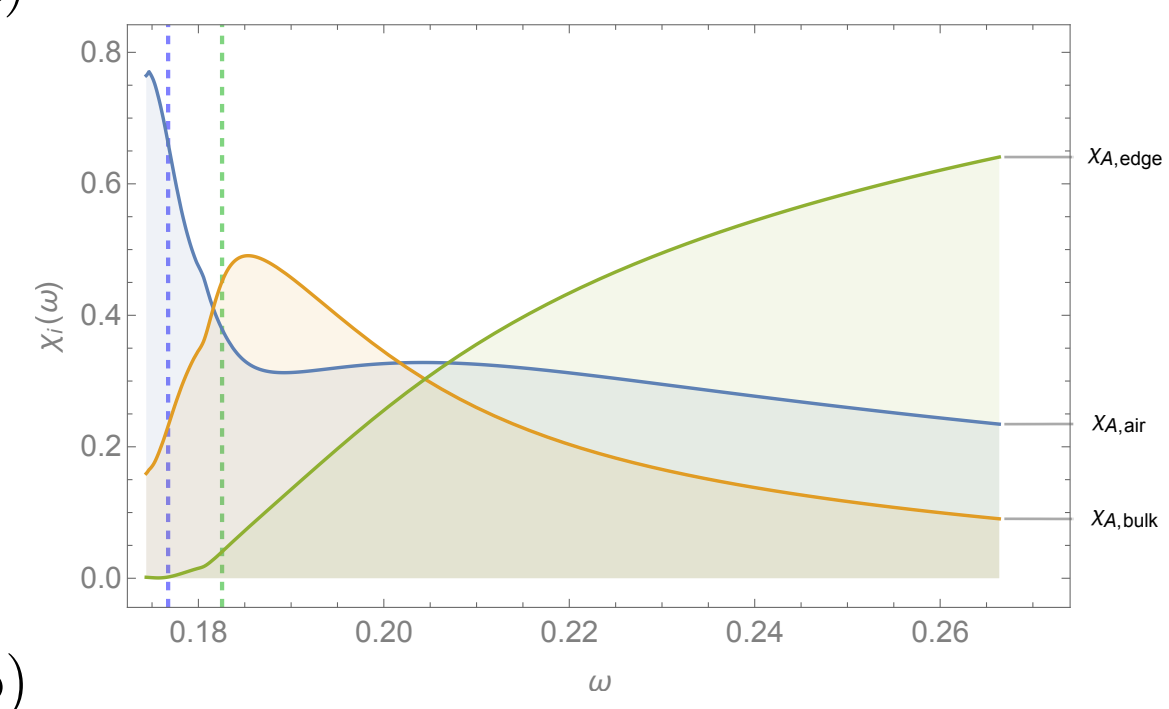

(b)

Figure S6: Localization fractions of electric energy in air, bulk, and edge regions for (a) the S mode and (b) the A mode. The vertical lines represent the cutoff frequencies for both modes, $\omega_{c,S} < \omega_{c,A}$.

mediated by guided bulk modes, which dominate propagation at intermediate frequencies—above the cutoff but not too high. This dominance is visible in the localization curves, where a bulk-dominant region satisfies $\chi_{\text{bulk}}(\omega) > \chi_{\text{edges}}(\omega)$ for $\omega \gtrsim \omega_{c,S}, \omega_{c,A}$, followed by edge dominance at higher frequencies ($\chi_{\text{bulk}}(\omega) \ll \chi_{\text{edges}}(\omega)$). This localization behavior corresponds to the energy-density patterns shown in Fig. 3 in the Main Text.

The previous figures establish a connection between the behavior of the dispersion relations for the S and A edge modes near their cutoff frequencies and the localization–delocalization transition. It is not coincidental that both effects occur in the same region. Fig. S2 shows that the dispersion relations of the S and A edge modes tend to merge at large values of k_y, or equivalently, large ω. In this regime, both dispersion relations tend to those of two almost uncoupled single waveguides modes at the L and R edges of the structure. The effective coupling between them can be evaluated by means of an integral of their overlapping tails. The guided modes of a single waveguide have exponentially decaying tails

of the form $\exp(-\gamma z)$ outside the core. Since the extinction coefficient γ is proportional to the frequency ω, it is easy to check that the overlapping integral between the L and R edge modes separated a distance l gives rise to an effective coupling of the form:

$$q(k_y, l) \sim \exp\left(-\frac{\omega(k_y)}{c} l\right). \tag{S22}$$

So that, either in the thermodynamic limit ($l \to \infty$) or in the high frequency limit ($k_y \to \infty$), the L and R modes become perfectly degenerated. In this regime, the symmetric (S) and antisymmetric (A) linear combinations of the L and R modes are also degenerate. Conversely, the gap between the S and A modes must be non-zero as the localization–delocalization transition is approached. In terms of the uncoupled L and R edge modes, this means that they can no longer be considered eigenmodes of the system. They must couple such that the true eigenmodes are the S (even) and A (odd) modes, which possess well-defined parity ($z \to -z$)—a symmetry of the SSH waveguide that must be satisfied by its eigenmodes. The origin of the coupling between the L and R edge modes in the transition region is evident in Fig. 3 in the Main Text: bulk modes dominate there, producing an effective coupling between the originally uncoupled L and R modes. At a fixed distance l, this coupling vanishes at large k_y or ω, and becomes only significant near the localization–delocalization transition.

This qualitative behavior can be described by a simple coupled-mode model for the L and R edge modes with an effective k_y-dependent coupling coefficient. We introduce the amplitudes $c_{L,R}(k_y)$, which define the uncoupled L and R edge modes, each with the same frequency dispersion relation $\omega_0(k_y)$, as they are degenerate by definition. The k_y and l dependent effective coupling between the L and R modes is represented by $q(k_y, l)$. The S and A eigenmodes then arise as the natural solutions of the eigenvalue equation

$$\begin{bmatrix} \omega_0(k_y) & -q(k_y, l) \\ -q(k_y, l) & \omega_0(k_y) \end{bmatrix} \begin{pmatrix} c_L(k_y) \\ c_R(k_y) \end{pmatrix} = \omega \begin{pmatrix} c_L(k_y) \\ c_R(k_y) \end{pmatrix},$$

which yields

$$c_S(k_y) = c_R(k_y) + c_L(k_y)\,,$$
$$c_A(k_y) = c_R(k_y) - c_L(k_y)\,,$$

with corresponding frequencies

$$\omega_S(k_y, l) = \omega_0(k_y) - q(k_y, l)\,,$$
$$\omega_A(k_y, l) = \omega_0(k_y) + q(k_y, l)\,.$$

From this model we infer that the mean value of the S and A mode frequencies is

$$\omega_0(k_y, l) = \frac{1}{2}\left[\omega_S(k_y, l) + \omega_A(k_y, l)\right],$$

and the effective coupling constant is given by the S–A frequency gap,

$$q(k_y, l) = \frac{1}{2}\left[\omega_A(k_y, l) - \omega_S(k_y, l)\right] = \frac{1}{2}\Delta\omega_{SA}(k_y, l)\,. \tag{S23}$$

The localization–delocalization transition can now be characterized by comparing the behavior of the S–A frequency gap with that of the edge-localization fraction. Figure S7 shows $\chi_{\text{edges}}(\omega, l)$ and $\Delta\omega_{SA}(\omega, l)$ plotted simultaneously. For the latter, we use $\omega = \omega_0(k_y, l)$ as a representative frequency at each k_y, providing a non-ambiguous definition when S and A modes are non-degenerate. The strong correlation between the S–A frequency gap and edge localization indicates that the transition can be characterized by this gap, or equivalently, through the effective coupling $q(\omega_0, l)$ via Eq. (S23). This interpretation enables this phenomenon to be understood in the framework of phase transitions and critical phenomena characteristic of condensed-matter physics.[8] In this context, the behavior of $q(\omega_0, l)$ in the delocalization region—smooth for $\omega > \omega_{c,S}, \omega_{c,A}$ and tending rapidly to zero for

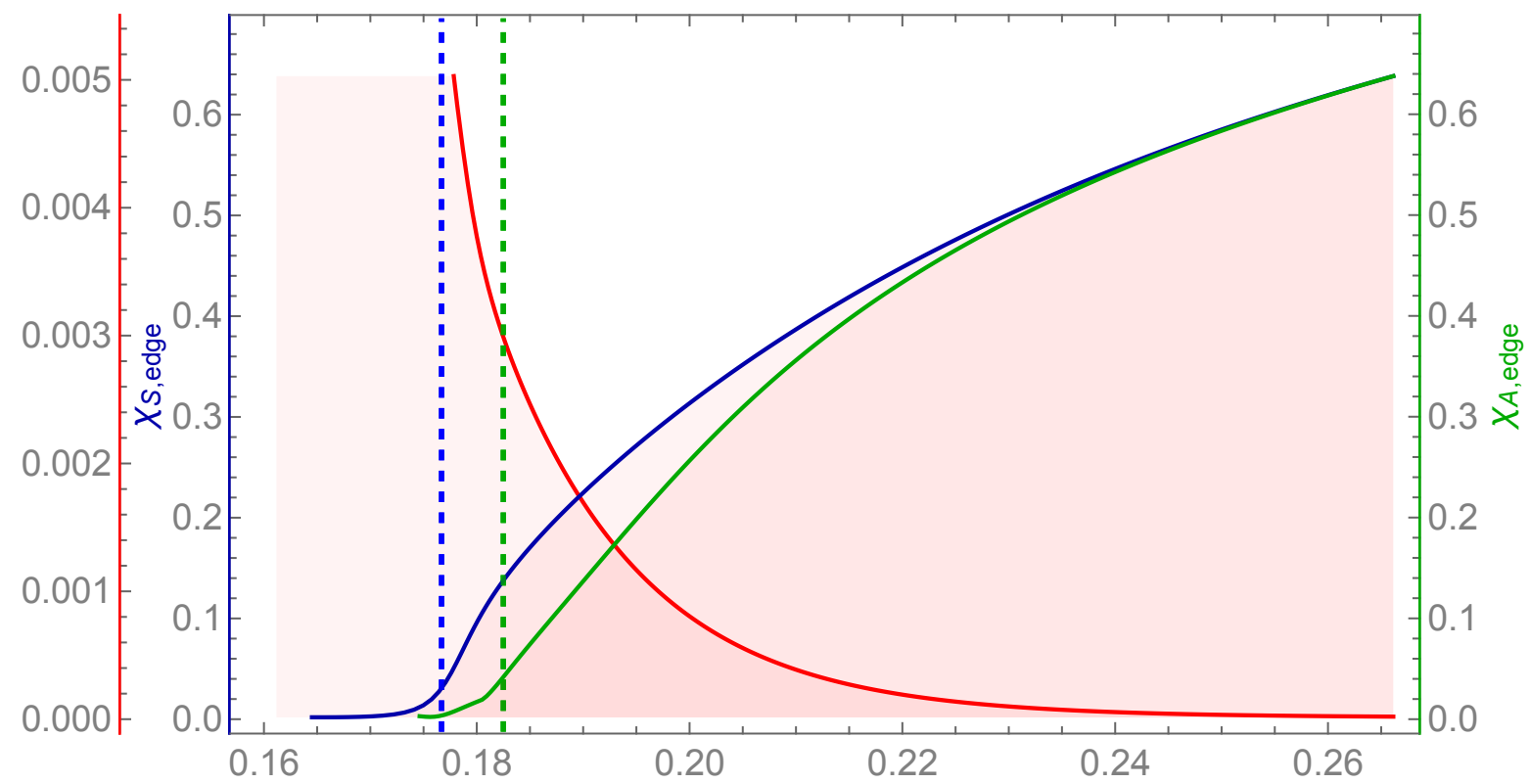

Figure S7: Edge-localization fractions for S (blue curve) and A (green curve) modes, together with the S–A effective coupling or frequency gap (red curve), as functions of ω. Vertical lines correspond to the frequency cutoffs $\omega_{c,S} < \omega_{c,A}$.

$\omega \gg \omega_{c,S}, \omega_{c,A}$—suggests that the localization–delocalization transition in an SSH topological photonic crystal can be regarded as a crossover phase transition.[9]

7 Effective 1+1 Hamiltonian model for L and R edge modes

The dynamics of the L-R coupled-mode model in the previous section is described by the following reduced k_y Hamiltonian in momentum space

$$\mathcal{H}(k_y) = \omega_0(k_y)(c_L^* c_L + c_R^* c_R) - q(k_y, l)(c_L^* c_R + c_R^* c_L) \tag{S24}$$

This Hamiltonian is the k_y component of the Fourier transform of the position-space Hamiltonian governing the 1+1 field amplitudes

$$\psi_{L,R}(y,t) = 1/(2\pi) \int_{-\infty}^{\infty} dk_y c_{L,R}(k_y) \exp[-i(\omega_{L,R}(k_y)t - k_y y)],$$

which describes the pulse evolution at each edge. For a narrow bandwidth pulse around a central frequency ω_0, the dispersion relation can be written as $\omega_{L,R}(k_{y,0} + \delta k_y) \approx \omega_0(k_{y,0}) +$

$\delta\omega_{L,R}$ in which $\delta\omega_{L,R} \ll \omega_0$ and where $k_{y,0}$ is the linear momentum associated to the pulse carrier frequency ω_0. The corresponding Hamiltonian in position space for such a pulse field has the form of a derivative expansion in the spatial coordinate y

$$H = \int dy \left[a_0 \left(\psi_L^* \psi_L + \psi_R^* \psi_R\right) - i\, a_1 \left(\psi_L^* \partial_y \psi_L + \psi_R^* \partial_y \psi_R\right) + a_2 \left(\partial_y \psi_L^* \partial_y \psi_L + \partial_y \psi_R^* \partial_y \psi_R\right) \right.$$
$$\left. - q_0 \left(\psi_L^* \psi_R + \psi_R^* \psi_L\right) + \text{higher order derivatives}\right] . \tag{S25}$$

This Hamiltonian corresponds to a dimensionally reduced field theory in one space and one time dimensions for the two component complex field $\Psi(y,t) = (\psi_L(y,t), \psi_R(y,t))$ enjoying internal phase symmetries directly linked to localization-delocalization mechanism under consideration. In this symmetry analysis the behavior of the coupling parameter q plays a crucial role. As we will see next, the reason behind this fact is that this Hamiltonian either in its momentum (S24) or in its position space (S25) representation exhibits different internal phase symmetries depending on the value of q. For convenience, we will use its momentum representation to perform our analysis.

For $q = 0$, the system is trivially invariant under independent phase transformations of the left (L) and right (R) modes, $c_{L,R} \to e^{i\theta_{L,R}} c_{L,R}$. Mathematically, this is expressed by the invariance of the Hamiltonian under the group $U_L(1) \otimes U_R(1)$ of unitary phase transformations. According to Noether's theorem, the corresponding conserved quantities (charges) associated with these L and R symmetries are $Q_L = |c_L|^2$ and $Q_R = |c_R|^2$, respectively ($\dot{Q}_L = \dot{Q}_R = 0$). Equivalently, one can define the vector and "axial" charges,

$$Q_V = Q_L + Q_R = |c_L|^2 + |c_R|^2 , \quad Q_A = Q_L - Q_R = |c_L|^2 - |c_R|^2 ,$$

which are both conserved when $q = 0$.

For $q \neq 0$, the $U_L(1) \otimes U_R(1)$ symmetry is broken by the presence of the coupling

term, leaving only the subset of transformations with $\theta_L = \theta_R$ as a valid symmetry of the Hamiltonian. Writing

$$\theta_L = \tfrac{1}{2}(\theta_V + \theta_A)\,,$$
$$\theta_R = \tfrac{1}{2}(\theta_V - \theta_A)\,,$$

where

$$\theta_V = \theta_L + \theta_R,$$
$$\theta_A = \theta_L - \theta_R,$$

one can verify by inspection that, for $q \neq 0$, the Hamiltonian remains invariant under θ_V transformations but not under θ_A transformations, which modify the phase of the coupling term:

$$(c_L^* c_R + c_R^* c_L) \to \left(e^{-i\theta_A} c_L^* c_R + e^{i\theta_A} c_R^* c_L\right)\,, \quad \text{when} \quad c_{L,R} \to e^{i\theta_{L,R}} c_{L,R}.$$

Thus, the symmetry-breaking pattern for $q \neq 0$ can be expressed as

$$U_L(1) \otimes U_R(1) \cong U_V(1) \otimes U_A(1) \overset{q\neq 0}{\hookrightarrow} U_V(1), \tag{S26}$$

which indicates that a non-zero coupling q induces the breaking of the $U_A(1)$ or "axial" symmetry of the system. This pattern reinforces the interpretation of q as the order parameter of a crossover phase transition between two phases with different internal symmetry properties, linked by the breaking pattern (S26).[8,9]

When $q = 0$, both Q_V and Q_A are conserved during propagation. In contrast, when $q \neq 0$, only the total vector charge Q_V is conserved, corresponding to the total power carried by the two edge modes. The axial charge Q_A is no longer conserved, as power is periodically exchanged between the edge modes through oscillations or beatings of the L and R topological

modes induced by the effective coupling q.

Of course, this symmetry breaking pattern also applies to the Hamiltonian in position space (S25), which describes the physics of a field theory that lives "on the y line", i.e., it is defined in $d = 1 + 1$ dimensions and has its L and R components as internal degrees of freedom. This feature permits to link this model to a rich set of field theories in $d = 1 + 1$ dimensions of great importance in condensed matter systems and also as testing models in particle physics. The Schwinger, Thirring and Sine-Gordon models are just some examples of this type of lower dimensional field theories. A good review of quantum field theories on the line can be found in chapter 7 of Tong's lecture notes.[10]

8 Optical dispersion parameters

Having rigorously established the conditions for the existence and propagation of the topological edge modes, we now examine their dispersive properties through the dispersion parameter–a key quantity describing group velocity dispersion (GVD).[11] Here, we consider only geometric dispersion, which depends strongly on design parameters such as the unit cell period Λ and the dimerization parameter Δ. As seen in Fig. 2 of the Main Text, a crucial aspect that can be modified by geometry is the slope of the propagation constant curve as a function of frequency, particularly in the region close to the cutoff frequency. For high frequencies, the dispersive behavior of the topological edge modes, regardless of the polarization, resembles that of the fundamental mode of the single waveguide (SWG). As the frequency decreases, however, the slopes become markedly different, until the cutoff frequency is reached, which in turn leads to a corresponding cutoff in the propagation constant. The geometric dispersion parameter is given by

$$D = -\frac{2\pi c}{\lambda^2}\beta_2, \tag{S27}$$

where λ is the wavelength and β_2 is the GVD coefficient.[11] Figure 4 of the Main Text shows how the geometric dispersion parameter varies with frequency for both TE and TM polarizations. Since two topological edge states are supported—each localized at one end of the finite structure—a splitting emerges in the dispersion curves at lower frequencies. This splitting separates the edge states into symmetric (in-phase) and antisymmetric (out-of-phase) modes. The distinction between these branches is clearly reflected in their dispersion characteristics for lower frequencies. At higher frequencies, both curves asymptotically approach that of the fundamental SWG mode.

The plots for D in Fig 4 corroborate the distinct features of topological edge modes for both TE and TM polarizations observed in the band structures of Fig. 2 when compared to the fundamental mode of a SWG near the cutoff region. Notably, we identify the positions of the zero-dispersion frequencies for TM polarization, where the SSH PhC waveguide transitions between normal and anomalous dispersion regimes. Such points are of particular importance for pulse propagation because they enable control over dispersion-driven broadening or compression effects. In the anomalous dispersion regime, for example, the balance between negative GVD and Kerr nonlinearity can support soliton formation.[11] More broadly, engineered zero-dispersion points underpin supercontinuum generation and enhanced nonlinear interactions in photonic crystal systems.[12,13] Although the present structure shows zero-dispersion crossings only for the TM polarization, we emphasize that the considerable design flexibility—including control over waveguide thicknesses, number of periods, and dimerization parameter—allows scenarios where the geometric dispersion parameter for TE-polarized edge states can also exhibit zero dispersion.

References

(1) Rylander, T.; Ingelström, P.; Bondeson, A. *Computational Electromagnetics*, 2nd ed.; Springer Science & Business Media, 2012.

(2) Jin, J. *The Finite Element Method in Electromagnetics*, 3rd ed.; Wiley-IEEE Press, 2014.

(3) Yeh, P. *Optical Waves in Layered Media*; Wiley-Interscience: New York, 1988.

(4) Lu, L.; Joannopoulos, J. D.; Soljačić, M. Topological photonics. *Nature Photonics* **2014**, *8*, 821–829.

(5) Ozawa, T.; Price, H. M.; Amo, A.; Goldman, N.; Hafezi, M.; Lu, L.; Rechtsman, M. C.; Schuster, D.; Simon, J.; Zilberberg, O.; Carusotto, I. Topological photonics. *Reviews of Modern Physics* **2019**, *91*, 015006.

(6) Asbóth, J. K.; Oroszlány, L.; Pályi, A. *A Short Course on Topological Insulators: Band-structure topology and edge states in one and two dimensions*; Lecture Notes in Physics; Springer, 2016; Vol. 919.

(7) Hasan, M. Z.; Kane, C. L. Colloquium: Topological insulators. *Reviews of Modern Physics* **2010**, *82*, 3045–3067.

(8) Altland, A.; Simons, B. D. *Condensed Matter Field Theory*; Cambridge University Press: Cambridge, 2010.

(9) Chalyi, O. V.; Zaitseva, E. V. The influence of dimensional crossover on phase transitions and critical phenomena in condensed systems. *Condensed Matter Physics* **2025**, *28*, 33501.

(10) Tong, D. *Lecture notes on gauge theory*; DAMTP, Cambridge, 2018; pp 334–387.

(11) Agrawal, G. P. *Nonlinear Fiber Optics*, 5th ed.; Academic Press: Boston, 2013.

(12) Ranka, J. K.; Windeler, R. S.; Stentz, A. J. Visible continuum generation in air–silica microstructure optical fibers with anomalous dispersion at 800 nm. *Opt. Lett.* **2000**, *25*, 25–27.

(13) Dudley, J. M.; Genty, G.; Coen, S. Supercontinuum generation in photonic crystal fiber. *Rev. Mod. Phys.* **2006**, *78*, 1135–1184.
